# Addressing the gap in health data management skills: an online self-guided course for researchers and health professionals

**DOI:** 10.1186/s12909-024-06405-y

**Published:** 2024-11-29

**Authors:** Naomi Waithira, Brian Mutinda, Kehkashan Shah, Evelyne Kestelyn, Susan Bull, Liam Boggs, Trudie Lang, Phaik Yeong Cheah

**Affiliations:** 1 Centre for Tropical Medicine and Global Health, Nuffield Department of Clinical Medicine, University of Oxford, Old Road Campus, Roosevelt Drive, Oxford, OX3 7FZ UK; 2 Mahidol Oxford Tropical Medicine Research Unit (MORU), Faculty of Tropical Medicine, Mahidol University, Nakhon Pathom, Thailand; 3 The Ethox Centre, Nuffield Department of Population Health, University of Oxford, Old Road Campus, Roosevelt Drive, Oxford, OX3 7FZ UK; 4 Faculty of Medical and Health Sciences, University of Auckland, Auckland, New Zealand; 5 Oxford University Clinical Research Unit (OUCRU), Hospital for Tropical Diseases, Ho Chi Minh City, Vietnam; 6 Health Data Research, Gibbs Building 215 Euston Road, London, NW1 2BE UK

**Keywords:** Data management, Data sharing, Data stewardship, E-learning, ADDIE, Kirkpatrick model, Health research, Training, Capacity building, LMIC, Low-resource settings, Data literacy

## Abstract

**Background:**

The healthcare sector is rapidly evolving with the rise of digital technology and data-driven decision-making. However, traditional medical education has yet to fully integrate training on managing health-related information, resulting in a significant skills gap among medical and research professionals. This gap is pronounced in low- and middle-income countries (LMICs), where data privacy concerns and inadequate infrastructure hinder efforts to utilise and share health data.

**Aims:**

To address this gap, we developed an online, modular course aimed at providing foundational skills on capturing, storing and sharing health data.

**Methods:**

The course was developed using the ADDIE(Analyze, Design, Develop, Implement, Evaluate) instructional design model. A needs assessment workshop involving 25 global health proffesionals identified key training gaps which informed the curriculum’s development. A multidisciplinary team from six institutions developed the modules. The course was piloted in a face-to-face setting with 37 participants and later adapted for online delivery via the Global Health Network platform. We evaluated the course using Level 1 of Kirkpatrick’s model for training evaluation.

**Results:**

Six foundational modules were developed: Introduction to Data Management, Data Quality, Data Repositories, Ethics of Data Sharing, Data Governance, and Costing for Data Management. Between December 2020 and April 2024, 6,384 individuals from 90 countries completed the course. Of these, 32% were from Africa, 15% from Asia, 16% from South/Central America and the Caribbean, and 24% from Europe. Summative evaluations, based on voluntary post-module surveys, demonstrated high relevance to participants’ learning needs (96.6%) and strong intentions to apply the skills gained (88.3%). Key motivators for enrollment included the course’s free access, relevance to professional or academic needs, and trust in the organizations and authors behind the content.

**Conclusions:**

The high enrolment and broad geographical reach demonstrates the potential of online training as a cost-effective tool to equip health practitioners and researchers with data literacy skills. Future evaluations will assess its impact on participants’ knowledge, behavior, and data-sharing and reuse practices.

**Supplementary Information:**

The online version contains supplementary material available at 10.1186/s12909-024-06405-y.

## Introduction

In recent decades, there has been a growing consensus among funders, regulatory agencies, and journals that de-identified individual-level health research data should be shared widely [1–3]. This consensus is driven by the rise in digital technology and the use of data in making health-related decisions at the policy level and in individual patient care [4]. 

With the outbreak of COVID-19 in early 2020, there were more calls for transparency and timely sharing of data to inform the global response [5, 6]. International funders, including the Wellcome Trust and the Bill & Melinda Gates Foundation, mandated that funding recipients must share data from the research they fund. However, the intent to share data [7, 8] and the volume of data shared remains lower than expected [9]. Additionally, authors seldom provide mechanisms for accessing individual participant data. Known challenges of sharing data from health research persist. These include complexities of broad consent [10], reliability of the data [11], worries related to data privacy and security [12], lack of trust [12], lack of data management infrastructure and capacity, including limited knowledge on how to share data [13, 14]. In addition, researchers may not be well informed on policies and guidance that enforce responsible data use. Many researchers, particularly in Low and Middle Income Countries (LMICs), have little training or experience in data management and sharing [13]. 

Furthermore, researchers working in low-resource settings have raised concerns that data sharing can potentially exacerbate existing inequalities between researchers in low-resource and high-resource settings. There is a fear that data collected in low-resource settings will be used in ways that do not benefit researchers who collected the data or communities that participated in the research [15–17]. 

Three strategies have been proposed as potential solutions to promote data sharing [18]. These strategies include investment in data systems and infrastructure, establishing incentive mechanisms to promote data sharing and the development of data science capacity [19]. Building upon the strategy of building data science capacity, this paper describes the design and development of a course aimed at enhancing competencies in the management and sharing of health-related data. At the time of conceptualisation and development of the course, few online and in-person courses were available, the available courses had limitations that included cost, a lack of focus on health-related data, and constraints in their relevance for health professionals.

## Objectives

We aimed to create a free-to-access, modular, and online course providing foundational skills on health data management to medical practitioners and researchers. The training is also suitable for research support professionals such as data managers, project managers, and statisticians. While the course predominantly focuses on quantitative data, the general principles are also relevant to qualitative data in health.

## Methods

**Fig. 1:**
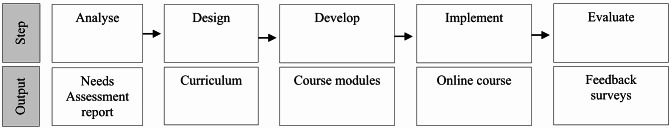
ADDIE instructional design framework steps and outputs

We developed the course using the ADDIE instructional design framework, a structured approach that tailors training programs to meet the needs of the learner [20]. ADDIE has been applied in developing medical courses to improve performance and promote behaviour change [21, 22]. The ADDIE framework involves a five-step process that includes analysis, design, development, implementation, and evaluation (Fig. 1). In this section, we describe the five steps of the ADDIE framework in the context of our course development.

### Step one: Analyse

In this phase, we determined the skill gaps and framed the learning outcomes through a training needs assessment(TNA) workshop. The workshop considered three-tiers of needs: organisational, operational, and individual. At the organisational level, we aimed to define the policies and governance mechanisms for institutions to fulfil their data-sharing mandates. At the operational level, we defined the requisite skills, knowledge, and infrastructure for efficient data management and sharing. Lastly, at the individual level, we described profiles of individuals responsible for data-related duties and identified relevant skills and knowledge. Participants worked in small groups to create ‘rich pictures’—a visual tool for portraying complex systems [23, 24]. They illustrated the data-sharing ecosystem, identifying key players, roles, and relationships. The workshop generated further data through presentations, activity sheets, and notes.

### Step two: Design

In 2019, a faculty was established, bringing together expertise in training, data management, data-sharing platforms, and ethics (Table 1). Representatives from collaborating institutions formed the core development team, responsible for assembling the curriculum, developing content, and delivering the course.

Based on insights derived from Step 1, the course’s structure and content were defined, learning objectives articulated, and instructional strategies constructed.

**Table 1:** Collaborators and area of expertise

Institution	Expertise
The Global Health Network (TGHN)	Training
Infectious Disease Data Observatory (IDDO)	Data sharing platforms, Data curation
The Ethox Centre	Ethics, Governance
Oxford University Clinical Research Unit	Training, Clinical research
Committee on Data of the International Science Council (CODATA)	Training, Data stewardship
Mahidol Oxford Tropical Medicine Research Unit (MORU)	Data management, Ethics, Governance, Costing

Referencing findings from a scoping exercise conducted by the European & Developing Countries Clinical Trials Partnership (EDCTP), we reviewed existing courses and resources on data management and sharing [25]. To avoid redundancy, modules that were comprehensively covered on other training platforms were removed from the provisional list. The team then refined high-level objectives for the remaining modules. Methods to assess the extent to which learners achieve intended competencies were agreed upon at this stage.

### Step three: Content development

Instructional materials, case studies, and exercises were assembled for each module based on existing literature and real-world experience from subject matter experts. To facilitate effective delivery in in-person settings, key concepts were summarised in PowerPoint slides and later transcribed in prose for the online course. The training material was put together by a lead developer, reviewed internally by the core team and then peer-reviewed by an independent subject matter expert. Feedback from the internal and independent reviews was incorporated before the module was finalised.

### Step four: Implementation

The course was piloted in a face to face setting prior to deployment electronically. Lead developers refined scripts for the online content along with supplementary material based on the feedback from the pilot phase. The content was then uploaded onto the online platform and disemminated on the Global Health Network [26] as well as via collaborating institutions’ social media platforms.

### Step five: Evaluation

To ascertain the course’s effectiveness and alignment with its intended objectives, we performed formative and summative evaluation based on level 1 of Kirkpatrick’s training evaluation model [27].

Formative evaluation was conducted during the design phase to verify whether the training gaps identified in the needs assessment were valid, whether the curriculum sufficiently addressed the needs, and to prioritise topics appropriate for a generalist, introductory course. Summative evaluation is ongoing. Learners are invited to provide feedback through a voluntary survey on completion of each module (Supplement 1). These responses will serve as a resource for future refinement. Future evaluations, based on levels 2–4 of the Kirkpatrick’s model will assess, ‘Learning’-whether learners’ knowledge or skillset improved after taking the course, ‘Behaviour’- examining the extent to which learners apply the skills in daily work and ‘Results’- the impact of behavioural changes on data management and sharing practices.

## Results

A training needs assessment(TNA) workshop in Bangkok convened 25 participants representing the course’s target audience and potential module authors. Clinicians, health researchers, data managers, statisticians, clinical trials experts and ethicists who primarily work in global health participated in the 3-day workshop. By the end of the workshop, a consensus was reached on key themes, and a provisional listing of course modules along with their objectives was developed (Supplementary 2). The learner profile, including prerequisite knowledge and skills for achieving learning outcomes, was defined. After reviewing existing resources [25] against the provisional listing of modules, 9 modules that were inadequately addressed in existing literature were selected and developed for the course (Table 2).

**Table 2:** Module listing at needs assessment and design phases

Topics defined during needs assessment	Topics after the design phase
• Introduction to data management • Data sharing requirements • Ethical considerations in data sharing • Study design and operations • Data quality • Data governance • Data re-use principles • Data repositories • Advanced concepts in data management • Data standardisation and anonymisation • Costing for data management and sharing • Qualitative data management • Sharing of data in public health emergencies	• Introduction to data management • Ethical considerations in data sharing • Data quality • Data governance • Data re-use principles • Data repositories • Advanced concepts in data management • Data standardisation and anonymisation • Costing for data management and sharing

In September 2019, the course was piloted in a three-day face-to-face training hosted by the Oxford University Clinical Research Unit (OUCRU) in Ho Chi Minh City. An international cohort of 37 individuals from diverse professional backgrounds, including medical doctors, pharmacists, nurses, biomedical technicians, clinical researchers, data managers and statisticians participated in the training. The lead developers delivered their respective modules with assistance from the course facilitators in the hands-on exercises. Materials used in the workshop are available on Zenodo [28]. 

**Fig. 2:**
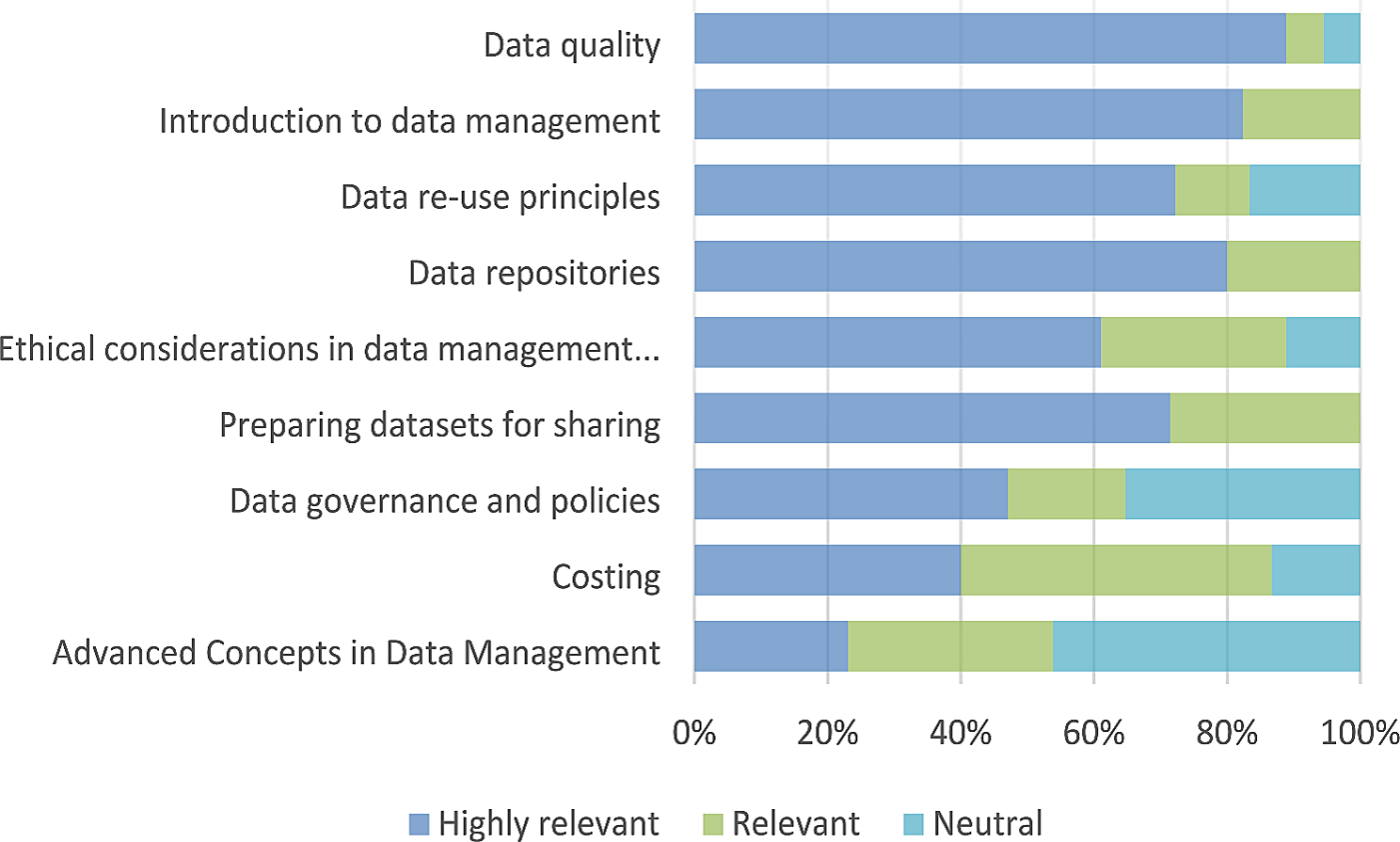
Ranking of modules by relevance

A formative evaluation aimed to verify whether the training gaps identified in the needs assessment were valid and sufficiently addressed by the curriculum was conducted via an online consultative workshop with researchers actively engaged in global health. Participants scored the modules based on relevance in a survey using a 5-point Likert scale. The ranking results are presented in Fig. 2. More than half of the participants deemed all modules relevant. Although considered appropriate, the module ‘Advanced concepts in data management’ was ranked lowest; some participants felt that the concepts were meant for ‘hardcore technocrats’ and less suitable for clinically oriented professionals.

Of the 9 proposed modules, 6 were deemed suitable for a generalist and introductory course, while 3 were considered ideal for an advanced level and, therefore, deferred for future implementation. An additional module, ‘Qualitative data management,’ was proposed for inclusion in future iterations. (Table 3)

**Table 3:** Listing of modules developed vs. modules for future development

Developed in Phase 1	To be developed in Phase 2
• Introduction to data management • Ethics of data sharing • Data quality • Costing for data management and sharing • Data repositories • Data governance	• Data reuse principles • Advanced concepts in data management • Data standardisation and anonymisation • Qualitative data management

The modules’ content was refined based on feedback from the pilot, and six modules were launched electronically on the Global Health Network eLearning portal between December 2020 and August 2023 [26] (Table 4). Although the modules are related, they need not be taken sequentially, learners can tailor the sequence of study to their individual needs by selecting specific modules in a self-directed manner. The estimated completion time for each module is between 45 min and 1 h. The learner can demonstrate their understanding of the content by taking a quiz on completion of the module. A certificate of completion is electronically issued when a minimum score of 80% is achieved, the quiz may be retaken if the score is lower than 80%. The online course is accessible free of charge to registered TGHN platform users, with a 2–3 min registration process.

**Table 4:** Learner’s occupation

Occupation	*N* (%)
Student	15.2%
Medical doctors	13.8%
Research Support Professional	13.1%
Public Health Professional	9.7%
Researcher	9.7%
Data manager	8.3%
Data entry/Fieldworker	7.6%
Pharmacist	4.8%
Research Participant	4.8%
Nurse	4.1%
Laboratory Staff	2.8%
Statistician	1.4%
Other (including EC/IRB member, Policy )	7.8%

## Course uptake

Between December 2020 and April 2024, 6,384 individual users completed at least one module. Of these, 32% were from Africa, 15% from Asia, 16% from South/Central America and the Caribbean, and 24% from Europe. Of the learners who completed the feedback survey, the majority identified as students(15.2%), doctors (13.8%), and public health professionals (13.1%). 10,019 module completion certifications were awarded, Table 5 shows a breakdown of the total number of certificates awarded and the number of learners who completed each module.

**Table 5:** Access metrics

Module Title	Launch date	Awarded certificates	Users
Introduction to data management	December 2020	3,898	5,666
Data governance, policies and Data Access Committees	February 2021	1,883	1,893
Data Quality	March 2021	1,712	1,993
Ethics of sharing individual-level health research data	July 2021	1,996	1,765
Costing for data management and sharing	December 2022	311	589
Data repositories	August 2023	219	264

## Evaluation

**Fig. 3:**
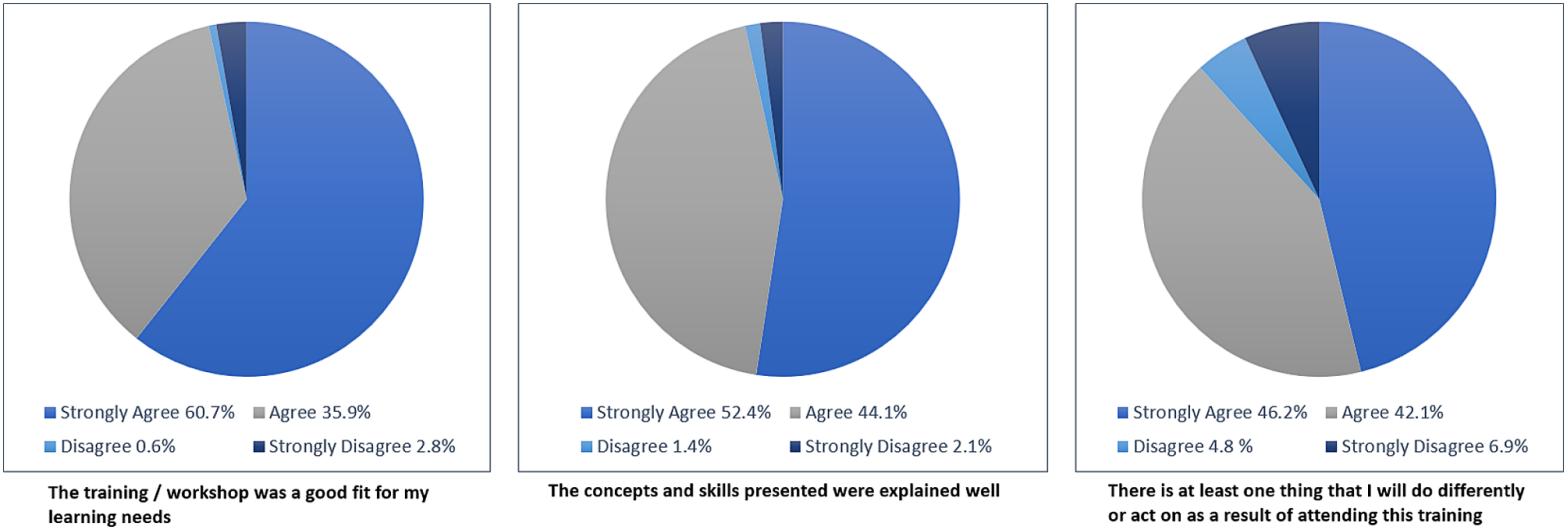
Feedback survey results

**Fig. 4:**
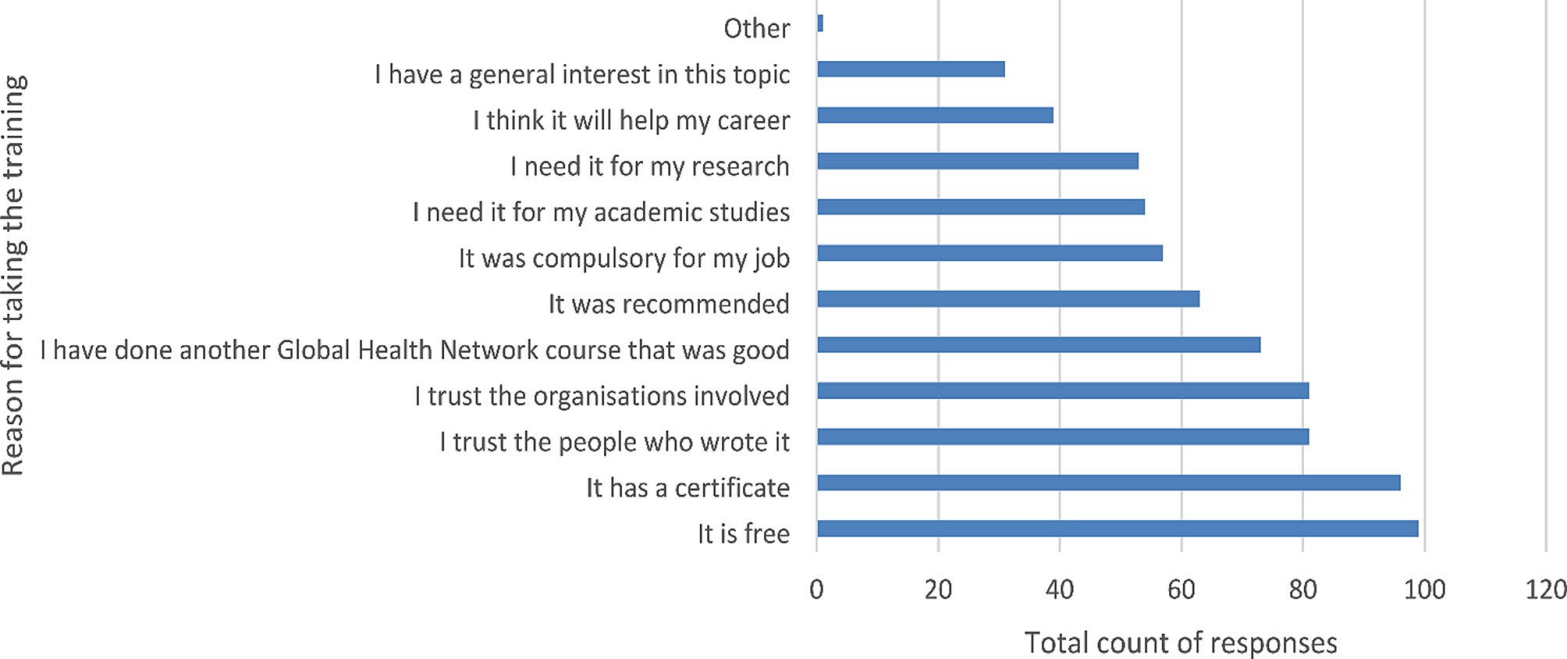
Reasons for choosing training. * participants could select more than one answer

In the ongoing summative evaluation, learners are invited to provide feedback through a voluntary survey on completion of each module (Supplementary 1). As at 30th April 2024, 145 survey responses had been received and these are summarized in Figs. 3 and 4.

## Discussion

We developed a foundational course on data management, tailored to the needs of healthcare professionals and researchers. The scope was defined through iterative consultation with prospective learners and subject matter experts resulting in a curriculum addressing practical and ethical considerations in capturing, storing and sharing health data. Our course provides an accessible entry point to those new to data management, specialised training in data science and software carpentry is available through forums such as CODATA [29] .

The uptake of the course confirms an interest in data literacy among health and research professionals. While the positive feedback from learners and demand for training is promising, it is essential to acknowledge that behaviour change is complex. As noted by Kirkpatrick [27], the effectiveness of training programs in promoting behaviour change is uncertain, and formal evaluation is needed to gauge their effectiveness in changing behaviour.

## Limitations

The online nature restricts interaction between the instructor and the learner, which potentially limits the depth of understanding compared to more traditional, face-to-face educational settings. Additionally, internet access remains a barrier to participation for individuals with limited access to internet. The low response rate to feedback surveys (2%), limits ability to assess the course’s effectiveness. Future evaluations must include strategies to encourage learners’ feedback.

## Future directions

The shift to online education has undeniably expanded access to information, but it has also highlighted the limitations of text-based learning in maintaining the learner’s engagement [30]. Incorporating video-based lessons and live webinars may enhance learner engagement and facilitate richer pedagogical exchanges. Translation to other languages and targeted dissemination strategies could broaden the reach to a diverse and global audience. Continuous refinement and structured evaluations are essential to maximize the program’s effectiveness and influence.

## Conclusions

While the COVID-19 pandemic highlighted gaps in data management and sharing skills, they are neither new nor unique to the health domain. Existing courses and resources on data management and sharing are fragmented across different platforms and are of varying quality, impeding the learners’ ability to access and benefit from them.

We address this gap by providing an accessible, peer-reviewed program tailored to the needs of healthcare professionals and researchers. Early feedback suggests a high level of relevance to learners’ needs. By making this program freely available, we aim to promote capacity building in resource-limited settings and establish a foundation for more robust and inclusive educational solutions in the future.

## Electronic supplementary material

Below is the link to the electronic supplementary material.

Supplementary Material 1

Supplementary Material 2

## Data Availability

Data generated or analysed during the development and evaluation of this course are included in this published article.
