# Supplementary material for: Addressing the gap in health data management skills: an online self-guided course for researchers and health professionals
Source: BMC Med Educ. 2024 Nov 29;24:1397. doi: 10.1186/s12909-024-06405-y (PMC11607898; doi:10.1186/s12909-024-06405-y)
Supplement: Supplementary file 1 — Supplementary Material 1 [file 12909_2024_6405_MOESM1_ESM.pdf]

# The Global Health Network feedback survey

## Page 1: The Global Health Network - Feedback survey

Please answer the following questions about the eLearning module / course you completed or event you attended.

Your feedback is important in helping us understand:

- To what extent this module or course is meeting your learning needs
- How the module or course could be improved for future participants
- What you feel you have learned

The findings from this survey may be published in a report, a scientific journal and/or presented at a conference. Your identity will remain anonymous in all publications and presentations of the findings.

This survey is also available in **portuguese**.

**Please Note**

The information that you supply in response to this training evaluation will be treated in accordance with the **University of Oxford's Data Protection policy**. The data collected in this form will be stored securely and will be only accessed by The Global Health Network. For more information please visit **The Global Health Network Privacy Policy** page.

For any other queries please email info@theglobalhealthnetwork.org

# The Global Health Network feedback survey

---

## Page 2: A few questions about you

Title of training course or event attended

Which of the following categories best describes your current role?

Please select

What type of establishment do you primarily work for / study at? ✱ *Required*

Please select

What organisation or institution do you primarily work for / study at?

Which country do you work in?

Please select

# The Global Health Network feedback survey

## Page 3: Your feedback

The training / workshop was a good fit for my learning needs

- Strongly Agree
- Agree
- Disagree
- Strongly Disagree

The concepts and skills presented were explained well

- Strongly Agree
- Agree
- Disagree
- Strongly Disagree

There is at least one thing that I will do differently or act on as a result of attending this training

- Strongly Agree
- Agree
- Disagree
- Strongly Disagree

What should we change or continue in this programme / workshop?

Change: *Optional*

Continue: *Optional*

Why did you choose this training? Choose all that apply.

- ☐ It is free
- ☐ It has a certificate
- ☐ I trust the people who wrote it
- ☐ I trust the organisations involved
- ☐ I have done another Global Health Network course that was good
- ☐ It was recommended
- ☐ It was compulsory for my job
- ☐ I need it for my academic studies
- ☐ I need it for my research
- ☐ I think it will help my career
- ☐ I have a general interest in this topic
- ☐ Other

If you would be happy to be contacted to provide further feedback, please complete the sections below (optional)

Name:

Email:
