# Supplementary material for: Addressing the gap in health data management skills: an online self-guided course for researchers and health professionals
Source: BMC Med Educ. 2024 Nov 29;24:1397. doi: 10.1186/s12909-024-06405-y (PMC11607898; doi:10.1186/s12909-024-06405-y)
Supplement: Supplementary file 2 — Supplementary Material 2 [file 12909_2024_6405_MOESM2_ESM.pdf]

Module title	Scope
Introduction	• What is research data, types of data and metadata<br>• What is data sharing, why share data<br>• Benefits and risks of sharing data<br>• Open access/open science/open data<br>• F.A.I.R data principles
Data sharing requirements	• Data sharing requirements by funding agencies and publishers<br>• Comparing data-sharing requirements by different stakeholders<br>• Data policies and regulations
Data Management I	• Developing a data management and sharing plan<br>• Data sources and methods of data collection<br>• Data curation and preparation<br>• Standardizing datasets<br>• Databases and storage technologies
Ethical Considerations	• Respecting participants and communities- informed consent, handling data on vulnerable populations<br>• Privacy and confidentiality<br>• Promoting equity for researchers, participants
Research set up	• Implications of data sharing on study design<br>• Data management and sharing provisions in protocols, Informed Consent Forms, CRF/Questionnaires
Data Quality	• Data validation<br>• Software and tools<br>• Monitoring and quality assurance<br>• Data quality metrics<br>• Compliance with guidance and regulation, e.g. GCP, FDA CFR Part 11, including audits and inspections
Data governance & policies	• Players: Data custodians, stewards, trustees<br>• Modes of data access: managed versus open access<br>• Data Access Committees- constitution and mandate<br>• Elements of an institutional data-sharing policy<br>• Monitoring compliance with an institutional policy<br>• Legal implications for researchers and research institutions in data sharing
Data re-use principles	• Research outputs- papers, source code, data<br>• Persistent identification of data outputs<br>• Data attribution and citation<br>• Monitoring re-use of data<br>• Selecting appopriate licenses for shared datasets<br>• Managing relationships in data sharing and reuse
Data repositories	• Digital repositories for health data<br>• Selecting a repository<br>• How to deposit data in a repository<br>• Data retention
Data Management II	• Data storage<br>• Backup and disaster recovery<br>• Integration of data systems<br>• Information Security Standards
Before pressing send (preparing data for sharing)	• Documentation<br>• Data standardisation<br>• Data de-identification/anonymisation techniques
Costing	• Costs associated with data curation, storage and sharing:<br>• Cost recovery options<br>• Budgeting for data management and sharing
